# ‘It made me feel part of the team, having my homework to do’ — women and specialist nurse experiences of remote follow-up after ovarian cancer treatment: a qualitative interview study

**DOI:** 10.1007/s00520-022-07470-z

**Published:** 2022-12-13

**Authors:** Fiona Kennedy, Leanne Shearsmith, Marie Holmes, Galina Velikova

**Affiliations:** Section of Patient Centred Outcomes Research, Leeds Institute of Medical Research at St James’s, University of Leeds, Bexley Wing, Beckett Street, Leeds, LS9 7TF UK

**Keywords:** Ovarian cancer, Follow-up, Electronic patient-reported outcome measures, Nurse-led telephone consultations, Qualitative

## Abstract

**Purpose:**

Ovarian cancer patients require monitoring for relapse post-treatment, and alternative follow-up pathways are increasing, which require in-depth exploration to ensure acceptability and inform implementation. This study aimed to explore women and specialist nurses’ experiences of participating in a feasibility study of an electronic patient-reported outcome (ePRO) follow-up pathway after ovarian cancer treatment.

**Methods:**

The feasibility study incorporated an ePRO questionnaire, blood test and telephone consultation with a specialist nurse, instead of face-to-face hospital visits. All women and the nurses involved were invited to take part in nested semi-structured interviews. Interviews were recorded and transcripts analysed using framework analysis.

**Results:**

Twenty interviews were conducted (16 out of 24 women who took part in the feasibility study and all 4 nurses). Four themes were identified: (1) readiness and motivators, (2) practicalities and logistics, (3) personal impact and (4) future role. An overarching theme highlighted how women strived to seek reassurance and gain confidence. Most women and nurses were positive about the ePRO pathway and would happily continue using it.

**Conclusion:**

This work provides invaluable insight into the experiences of women on remote ePRO follow-up post-treatment. Important logistic and implementation issues were identified, which should inform future large-scale work to introduce and evaluate remote ePRO methods in cancer follow-up. This work highlights the key factors influencing women’s readiness and acceptability of an ePRO pathway, and how services should be carefully designed to ensure patients feel reassured and confident post-treatment. Furthermore, it highlights that flexibility and patient preference should be considered in remote service delivery.

**Trial registration:**

ClinicalTrials.gov ID: NCT02847715 (first registered 19 May 2016).

**Supplementary Information:**

The online version contains supplementary material available at 10.1007/s00520-022-07470-z.

## Background

Around 7500 annual ovarian cancer (OC) cases are diagnosed in the UK [1], and over 54,000 women in England are estimated to be living with and beyond OC in 2019 [2]. Relapses are common [3], and therefore standard practice is face-to-face follow-up for 5 years, alongside serum biomarker testing (CA125), physical examination and imaging [4].

Services are under pressure to support/monitor those living with and beyond cancer, and alternative follow-up services are increasing [5, 6], including patient-initiated follow-up (PIFU) where patients are only seen if they initiate contact [6]. Various studies, both qualitative and randomised controlled trials, have illustrated positive experiences/outcomes and acceptability of nurse-led telephone follow-up amongst gynaecological (including ovarian) cancer patients [7–10]. This trend is likely to grow further in light of the coronavirus pandemic which necessitated the rapid introduction of remote-based services [11] and evidence of the acceptability of such services [12, 13]. The NHS long-term plan emphasises personalised care planning, stratified follow-up and using digital technology [14, 15]. One approach is through patient-reported outcomes (PRO, defined as patient’s own assessment of their symptoms and health [16]), which can be assessed through self-reported paper or web-based questionnaires. PRO have been found to positively influence communication, quality of life and survival [17–22]. In OC, most previous research exploring the use of PRO has been with patients on active treatment, with few studies during follow-up [4, 23]. The studies that have focused on the follow-up period, mostly in other cancers, have often used PRO/electronic PRO (ePRO) in addition to routine visits [20, 23–26], rather than as an alternative.

The **e**lectronic **P**atient self-**R**eported outcomes to **I**mprove cancer **M**anagement and patient **E**xperiences (ePRIME) system was composed between 2016 and 2018 following robust information technology and development work [27]. Qualitative interviews with clinicians and women previously treated for OC were conducted in 2016–2017 to explore their views of current follow-up and the prospect of ePRO follow-up [28]. The resulting system and pathway (described below) informed by this preliminary work sits in-between PIFU and routine clinic visits [28]. The 12-month feasibility study recruited 24 women treated for OC [29] and showed high compliance with ePRO questionnaires and community bloods, fewer visits, more phone calls (as planned) and high patient satisfaction. However, the importance of exploring new innovations using qualitative methods has been emphasised [30] to inform intervention refinement and future implementation strategies. This study aimed to explore the experiences of the ePRO follow-up pathway amongst women treated for OC and nurses.

## Methods

### Design and participants

This nested qualitative study utilised a pragmatic qualitative descriptive approach [31], which is commonly used in healthcare research to provide straightforward descriptions of experiences and perceptions [32]. The wider feasibility study pathway incorporated 3-monthly ePRO symptom reporting (with linked on-screen advice to the patient or auto-generated emails based on a clinically developed algorithm), community blood tests and a nurse-led telephone review (with access to the ePRO responses). Full eligibility for the women who took part is specified in Kennedy et al. [29]. Women had completed their OC treatment, and their clinician agreed they could be followed remotely post-treatment. Women had to have access to the internet in order to complete the ePRO questionnaires (confirmed through a Delphi process [27]) and holistic needs (e.g. emotional, family, finances). Women with overt psychopathology/cognitive dysfunction or requiring face-to-face appointments (e.g. on clinical trials or maintenance treatment) were excluded.

The overall study received ethical approval (UK Health Research Authority Research Ethics Committee) and local approval to run at two hospitals (a large cancer centre and smaller hospital) from September 2018 to November 2020. There was no pre-specified sample size for this qualitative study. Attempts were made to reach all women, including those who had relapsed, chosen to withdraw or not engaged fully (e.g. no ePRO completions); hence, it was a purposive (had experience of the ePRO pathway) and self-selected sample as women could choose whether to participate in the interviews. All specialist nurses who had supported the wider ePRO feasibility study by undertaking the 3-monthly telephone reviews were also invited to participate in an interview.

### Data collection

Women were invited to the interviews at the end of their feasibility study involvement by the lead researcher (FK). All interviews were conducted from October 2019 to November 2020 by female researchers who were experienced in qualitative research and had been involved in the feasibility study (predominantly one research fellow/Ph.D. (FK), two research assistants/MSc (MH) and BSc (LS)). Semi-structured interview schedules were developed by the research team (including a patient representative) covering core topics relating to experiences of the ePRO pathway (see Online Resource file S1).

### Procedure

Written informed consent was provided by women at the start of the feasibility study, after they had received/read a participant information sheet. This included consent to digitally audio-record any end-of-study interviews and use any anonymous extracts. Most interviews were telephone-based due to COVID-19 restrictions, and consent to audio-record was verbally re-confirmed before the interview commenced. Interviews were transcribed verbatim, pseudonyms used to distinguish participants and identifying features removed.

### Data analysis

NVIVO 12 facilitated the data storage/organisation, and transcripts were explored collectively (patients and nurses) to enable an overall picture of views. Framework analysis [33] was chosen as it allows both a deductive and inductive approach to code development [34], which was appropriate for this study where specific pre-defined aspects to the ePRO components were explored, but inductive aspects were also sought. The five stages of framework analysis were followed: familiarisation, developing a thematic framework, indexing/labelling, charting, mapping and interpretation. Firstly, the transcripts were read several times and deductive (e.g. value of the core features) and inductive codes developed. One researcher (FK) initially analysed all transcripts, but the rigour of this stage was supported by another researcher (MH) independently coding 20% of transcripts (2 women/2 nurses), followed by discussion/agreement on the final thematic framework and interpretation. Charting is a process for summarising and synthesising the data to identify thematic links, and this was carried out using the NVIVO framework matrix feature. Mapping and interpretation involved exploring the associations across the data as a whole, and this process was facilitated by the use of diagrammatical representations of the themes [33].

## Results

Out of 24 women who took part in the feasibility study [29], 16 women participated in the interviews and the 4 specialist nurses who supported the ePRO pathway. Interviews lasted between 5 and 68 (women) and 12–45 min (nurses). Four of those not interviewed had relapsed, and four had withdrawn (note, two of withdrawals were on study < 3 months with no ePRO completions). Table 1 illustrates the interviewee characteristics. Six women were recruited less than 12 months post-treatment, whereas ten were 16–107 months post-treatment.

**Table 1:** Characteristics at overall study entry of the 20 interviewees (*n* = 16 women; *n* = 4 nurses)

ID	Age (years)	Time since last treatment end^a^ (months)	Treatment summary (first or second line)	Study status at interview (women)/length of time working in OC (nurses)
Women (*n* = 16)				
PT1^b^	62	3	First line	Completed 12 month
PT2^b^	60	1	First line	Relapsed at 12 months
PT3^b^	75	2	First line	Completed 12 months
PT4	59	38	First line	Completed 12 months
PT5	53	23	First line	Completed 12 months
PT6	78	32	First line	Relapsed at 10 months
PT7	51	16	First line	Completed 12 months
PT8	55	4	Second line	Completed 12 months
PT9	21	28	First line	Completed 12 months
PT10	69	41	Second line	Completed 12 months
PT11	42	18	First line	Completed 12 months
PT12	66	1	First line	Completed 12 months
PT13	54	37	First line	Relapsed at 12 months
PT14	55	107	First line	Did not complete
PT15	78	19	First line	Did not complete
PT16	76	3	First line	Did not complete
*Overall summary*	*Median 59.5; quartile 1* = *53.75; quartile 3* = *70.5*	*Median 18.5; quartile 1* = *3; quartile 3* = *33.25*	*First line* = *14 (87.5%)* *Second line* = *2 (12.5%)*	*Completed 12 months* = *10* *Relapsed* = *3* *Did not complete* = *3*
Nurses (*n* = 4)				
Nurse 1	Not recorded	–	–	Over 10 years
Nurse 2	Not recorded	–	–	Over 10 years
Nurse 3	Not recorded	–	–	Under 5 years
Nurse 4	Not recorded	–	–	Over 10 years

– Not applicable

^a^At recruitment/entry to the overall feasibility study

^b^Most interviews (except *n* = 3) took place during/after the first UK coronavirus pandemic lockdown in March 2020

**Fig. 1:**
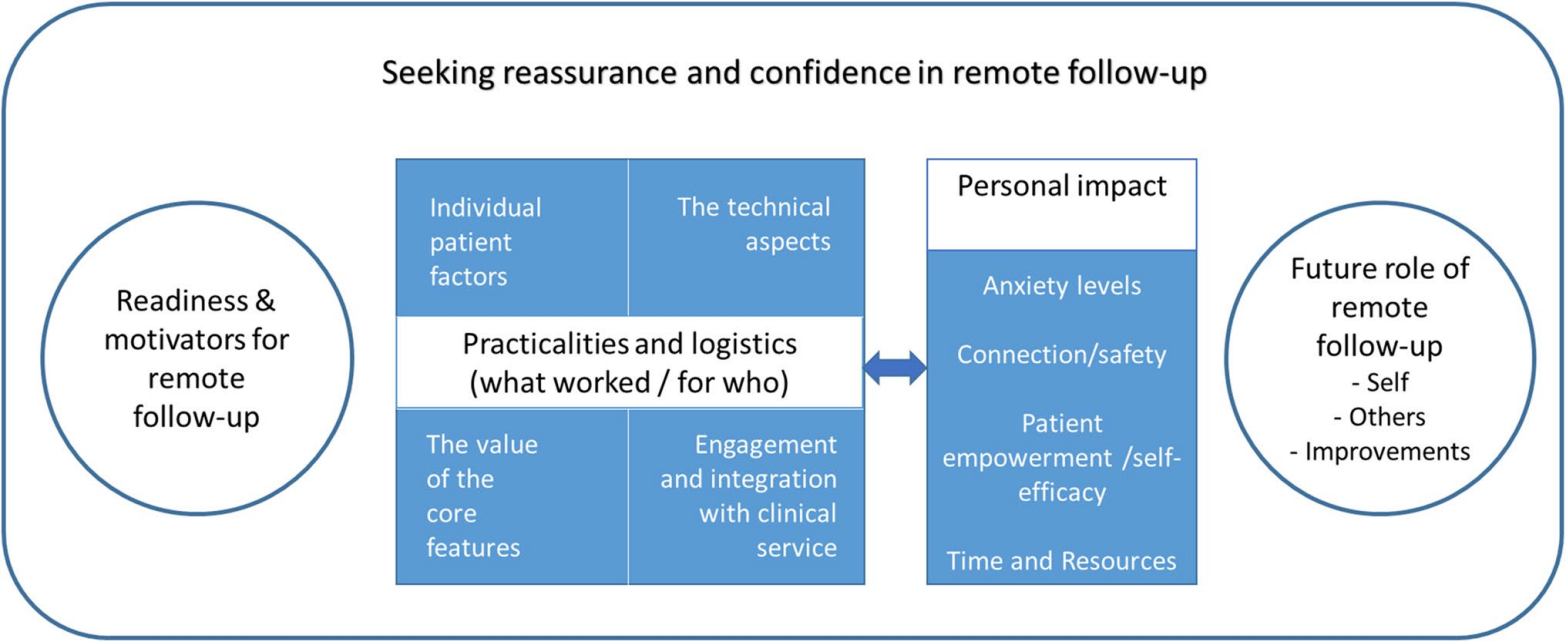
Overarching themes

Four themes emerged (readiness and motivators for remote follow-up, practicalities and logistics, personal impact, future role), which are depicted in Fig. 1, and an overarching theme identified illustrated that experiences of ePRIME were centred on the need for women to seek reassurance and gain confidence that they were still disease free. Additionally, as further described below, there was a key relationship between practicalities/logistics and personal impact themes, which is represented by the bi-directional arrow in Fig. 1. Each individual theme is discussed, example extracts provided, with further extracts in Online Resource file S2.

**Fig. 2:**
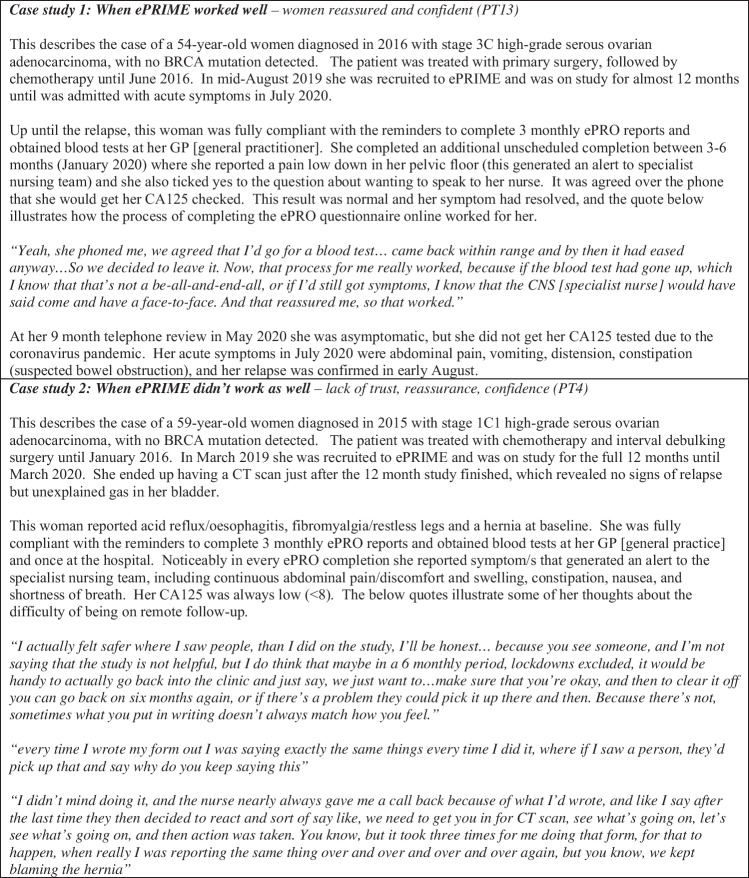
Case studies of two women on ePRIME, which illustrates the overarching theme ‘seeking reassurance and confidence in remote follow-up’

Furthermore, Fig. 2 highlights the overarching theme by presenting two case studies showing the real-life contrasting experiences. When the pathway worked well (technical system + obtaining blood tests + support/contact with clinicians), this resulted in feelings of reassurance, confidence and positive impact. In contrast, if the pathway worked less well, women felt a lack of trust, confidence and uncertainty in their follow-up.

### Theme 1: Readiness and motivators for remote follow-up

This theme reflects the study entry considerations, including motivations and views both women and nurses had on factors influencing individual readiness to accept remote follow-up.

#### Motivators for joining

Personal motivators included convenience of not attending hospital-based appointments and for some the personal stress and anxiety of the hospital environment. Others were reassured by their consultant’s endorsement, felt the face-to-face visits had become inefficient, and the open-access ePRO system enabled them to self-monitor whist still getting support:

It’s like a cushion for me, it meant that I could access it any time. (patient (PT), PT13).

Others described wanting to support research, improve services for future patients, altruism and wanting patients who needed face-to-face appointments to receive the support:

So you just feel like you’re wasting a lot of people’s time when there’s other people who could probably do with a bit more time. (PT11)

#### Patient readiness

Both women and the nurses emphasised the readiness they considered essential before entering a remote pathway. Needing to ‘feel well’ both physically and emotionally was emphasised, as well as being ready to move on:

while you’re well and trying to get on with your life, it’s best to have phone calls, it’s best to keep you out of hospitals. (PT12)

Views about the timing of introducing remote follow-up varied, but generally, face-to-face visits were valued during the first year post-treatment. However, Table 1 shows the variety in months since treatment. Some who joined immediately post-treatment found this acceptable, and some who joined further post-treatment would have accepted earlier.

### Theme 2: Practicalities and logistics of remote follow-up

#### Individual patient factors

Most women were reasonably computer competent, but some needed support or in one case the women’s husband did all the computer completions alongside her. Secondly, some women felt remote follow-up may be more suitable for those whose cancer is lower grade or more accurately monitored by blood tests. The complexity of existing symptoms and the presence/absence of comorbidities were also highlighted. Some women logged very few symptoms, or only around the time of relapse. In contrast, a few reported symptoms throughout or struggled to report their symptoms confidently due to complexities of disentangling other comorbidities versus cancer-related symptoms (Fig. 2—case study 2):

I haven’t just got one thing going on, it’s all a bit muddled, it’s a bit like a muddy pool…if someone just had the cancer and nothing else I think that that would be a lot better (PT4)

#### The technical aspects

The system was easy to use and the automated email/text reminders useful, but a few women had technical issues accessing the system or with reminders, which affected their confidence:

the unfortunate first time, when I had some symptoms, it worried me when the system had crashed. (PT1)

Furthermore, three women who did not engage with the 3-monthly ePRO completions (see feasibility results [29]) did not remember receiving any reminders.

The specialist nurses were positive about accessing and reviewing patient’s ePRO symptoms, but the lack of regular use meant they did not always feel competent:

I think because it was so ad-hoc, you forgot how to do it (Nurse 2)

Furthermore, the two hospitals differed in the level of technical integration into clinical systems (e.g. only one hospital had automatic linkage into the electronic patient record, EPR), and where full EPR integration occurred, the nurses were more positive.

#### The value of the core features

Most women found the open-access ePRO questionnaire highly valuable, emphasising it was easier to complete this thoroughly in their own time, and it was more detailed than their previous face-to-face follow-up:

you sort of sit in front of the nurse, and she’s asking you similar things, but I thought the ePRIME thing was probably a little bit more in-depth. (PT7)

However, a few women found it difficult when their symptoms did not fit neatly into the questions, and despite a free-text ‘other symptoms’ section, this was perceived as more difficult than talking directly to a clinician. The nurses had contrasting views, with some seeing significant added benefit of viewing the ePRO data upfront versus seeing less value if they still needed to telephone the patient (Online Resource S2, quote 25–26):

before you ring that patient you’ve got a plan in your head, you’ve got some suggestions you can make (Nurse 4)

Some women were impressed with the speed of the contact if they reported a symptom, prompting blood tests and/or discussions with clinicians (Fig. 2—case study 1). A few women generated alerts for ongoing symptoms or symptoms related to another illness, which was less helpful (Fig. 2—case study 2). On-screen advice for mild symptoms was useful for some, but not for others who already knew what to do. The nursing team reflected the judgements they made when receiving an alert (i.e. call straightaway or wait for upcoming telephone review, see quotes 31–32). None of the nurses had an issue with the volume of alerts, but one suggested they could be refined further to reflect symptom changes overtime.

Access to blood tests was relatively straightforward, but a few women reported difficulties (e.g. one doctor’s surgery did not take bloods, living out-of-area), and some experienced delays/confusion receiving the results. Nurses mentioned the process of sending blood forms as one of the logistics potentially adding to their workload.

One nurse theorised that future remote-based services could take bloods without the telephone review appointments. However, women were reassured by the call, specifically emphasising they valued receiving the CA125 result and ‘it’s extra reassurance when they ring up’ (PT2).

#### Engagement and integration with the clinical service

The nurses’ engagement with the pathway and views of workload, administration and integration with the clinical service appeared influential, with some resistance and negativity around the change from usual practice. At both hospitals, the nurses had some concerns about the workload and administration required (Online Resource S2 quote 38–39). Some emphasised the small nature of the feasibility study, and that broader use would embed the processes. More positivity was evident amongst nurses based at the hospital where the system was integrated into the EPR, and the research team and specialist nurses were based in the same building — enabling a higher level of support.

### Theme 3: Personal impact

#### Anxiety levels

Many women (and nurses) reported reduced anxiety and stress compared to attending the outpatient clinic:

a couple of times when they, I prompted them to give me an appointment…Although it’s great to know that that, of course, can happen when it needs to, but it’s, I found the telephone system less, made me less anxious. (PT1)

Some reflected that they were less likely to forget the issues they wanted to discuss:

a questionnaire in your own home when you’re relaxed and you, you know, you’re not going to miss things (PT5)

#### Connection and safety

Feeling connected and in-touch with the service was discussed in contrasting ways. Many women felt the open-access ePRO enabled them to still communicate efficiently, resulting in a feeling of safety (Fig. 2):

knowing I could fill that in any time I wanted was reassuring, strangely. I knew that if I wanted to, I could think about and note my symptoms every day and have a graph [laughs]. That made me feel strangely safe. (PT1)

However, some women ‘did miss the personal contact’ (PT3), felt there was something lacking in remote follow-up and discussed a hybrid approach:

I’d prefer, every so often go to the hospital and have face-to-face…Yeah, a bit of a mixture. (PT10)

#### Patient empowerment/self-efficacy

Some felt the ePRO pathway increased their involvement and awareness of symptoms to monitor:

I was empowered to fulfil my role…I’m in the team as well and the doctors and nurses themselves are in the team, and we work together. And it made me feel part of the team, having my homework to do. (PT1)

Similarly, the nurses felt they often traditionally took on the responsibility for monitoring patients during follow-up, and ePRIME shifted the responsibility: ‘it has really empowered the patient, because it’s made the health professional take a step back and we’re not good at that’ (Nurse 4).

#### Time and resources

Saving time and resources were frequently discussed, both in terms of women not having to come to hospital in-person when well (especially younger, working or those who lived further away) and the time being available for those who need it. Furthermore, some nurses felt that viewing the ePRO symptoms in advance of the telephone review ‘was a really good overview, and also helped shorten the consultations’ (Nurse 4). In contrast, the ePRO pathway administration was highlighted, and one nurse felt it created additional workload.

### Theme 4: Future role of remote follow-up

The future of remote follow-up and potential improvements if ePRO follow-up became mainstream were discussed.

#### Future use

Seven women were very clear they wanted to continue on ePRO and two wanted telephone follow-up alone (having not used the ePRO). Two women emphasised being happy with ePRO when well and cancer free (one had relapsed), and one stated:

I don’t think I’d do it without the electronic needs assessment…because they get the information before the phone call…the eHNA absolutely gives you time to think about those before you’re having the actual conversation. (PT13)

Finally, four women discussed preferring a hybrid approach — being seen every 6 months or annually.

Most women would recommend ePRIME to other patients, although personal circumstances were mentioned in terms of ‘if well’, ‘working’ and ‘had/could use a computer’.

Nursing staff had mainly positive views about future remote follow-up services. One nurse was very passionate of it being rolled out in her telephone nurse-led clinics. The other three nurses had more muted views suggesting that consultant-led follow-up should continue initially (as OC patients often relapse within 18 months), but recognising that in a post-COVID era it was likely that telephone-based patient self-management would be increasingly used.

#### Suggestions for improvements

Various improvements (Table 2) were suggested for future remote follow-up, in order to enhance women’s reassurance and confidence with the process. The most common were video consultations, improved facilitation, access and communication around blood tests/results and more availability for ePRO free-text comments.

**Table 2:** Areas for improvement suggestions by women and specialist nurses mapped to the main themes

*Readiness and motivators* • Use during treatment (women and nurses) • Use for all patients prior to telephone review (nurses) • Hybrid (combination of face-to-face and ePRO) approach (women)
*Practicalities* • Questions — More opportunity for free-text comments, adding own question and being reminded of it on subsequent completions; add a generalist health question (women) • Facilitation/access and communication around community blood test/results (e.g. cover note for women to give to general practitioner; earlier bloods reminder; improved communication about blood results; more guidance on where can get bloods) (women) • More communication/feedback that clinical team have reviewed results (women) • More direction/specific instructions to access website for ePRO completions (women) • Improved reminder timing (women); instead of relying on reminders, tell patients to do just prior to telephone review/same day they get blood test (nurses)
*Ideas for future use* • Video consultations (women and nurses) • Use an alternative to CA125 (e.g. scans) for those whose cancer does not show in blood (women) • Links to peer support within ePRO questionnaire (women) • Change to an ad-hoc symptom alert app (nurses) • Enable ePRO system to generate document/letter to general practitioner to reduce workload (nurses) • More sophisticated alert system — i.e. comparing current to previous symptoms (nurses)

## Discussion

The in-depth accounts of 16 women who participated in the ePRIME feasibility study provide a unique insight into their experiences on the remote monitoring pathway. To the best of our knowledge, this is the first qualitative study exploring this amongst women after OC treatment. Furthermore, interviewing the four nurses who supported the pathway provide insights into wider implementation issues. This work illuminates the feasibility results presented in Kennedy et al. [29] by highlighting factors that influenced women’s readiness for a remote-based pathway and the importance of building an ePRO service that continues to support and reassure women. This maps onto previous evidence highlighting the importance of individual’s experiences and preferences of technology use in cancer care and implementation approaches for PRO integration and sustainability in routine care [35, 36].

Women had to be physically and emotionally well for ePRO follow-up, but could reach this point at different times post-treatment. Some women were keen immediately post-treatment, whereas others wanted face-to-face follow-up, especially during the first year. Linking to the overall feasibility results [29], around one-third (17/48) of eligible patients declined to take part due to wanting face-to-face follow-up. However, a one-size-fits-all time point may not be appropriate, and instead, it should be guided by patient preference and clinician expertise. Furthermore, Kennedy et al. [29] highlight how a number of women were not eligible for the ePRO pathway due to being on new maintenance treatments requiring face-to-face visits (e.g. niraparib) or clinician decision (e.g. non CA125 secreting). Since the recruitment to this study, the coronavirus pandemic has prompted more patients to engage with remote-based/telephone follow-up, which may have influenced both clinician and patient future acceptance [37, 38], and requires exploration.

For many women, the ePRO pathway provided good connection/support, without the anxiety of attending hospital, and they were keen to continue. Similar themes have been evident in previous studies exploring nurse-led, telephone follow-up amongst individuals after gynaecological cancer [8, 9, 39] and ePRO use during active treatment [36]. This study adds to the growing evidence of the effectiveness of specialist nurse-led follow-up [7–10] and highlights the different methods of follow-up (e.g. telephone, ePRO approaches) that could be facilitated by nurses in clinical practice. The current study also showed a link between the practical logistics of ePRO pathway and the resulting personal impact. Women who had cancer that was more complex (e.g. non-secretors of CA125) [40], other comorbidities or low technology/computer confidence were less likely to feel confident and reassured. However, willingness and confidence with ePRO methods may increase if patients first had access alongside their face-to-face appointments or during treatment to gain trust with the system. Remote follow-up work in prostate cancer have utilised an initial self-management workshop at the start of remote follow-up [41], which could be valuable in OC. Finally, ePRO follow-up may work most effectively by excluding those individuals with complex disease or symptomatic profiles, illustrating the importance of risk-stratified pathways [42].

There were some reports of ePRO pathway difficulties (e.g. community bloods, electronic reminders, staff administrative issues/ workload), highlighting the need for ePRO services to be properly resourced and supported internally (e.g. integration into EPR) and externally (e.g. primary care collaboration) [40, 43]. In relation to this, future research should also prioritise economic evaluations of remote-based follow-up, which has been rarely studied in large-scale studies [44, 45].

The main study limitations are the self-selecting nature of the women who took part and the specific nature of the wider feasibility study. The views are only from a sub-set of women after OC from two hospitals who were willing to try ePRO follow-up, and then only a self-selected sub-set interviewed [46]. The local context and existing relationships with the clinicians are likely to have influenced experiences. Furthermore, the interviews were conducted by members of the research team who were involved in the feasibility study, which could have prompted more positive responses. Similarly, none of the women who withdrew were interviewed. The findings may not be generalisable to the wider representation of OC patients in the UK — for example, interviewees ranged from 21 to 78 years old, and only 4 (25%) were the age of typical OC incidence (75–79 years) [1]. Therefore, this sample was likely skewed towards younger, working age women who were computer savvy, and therefore, more work is needed to explore how age influences ePRO acceptability and its suitability in older patient cohorts. Future research should also explore withdrawal reasons and whether adaptations could increase ePRO follow-up acceptability (e.g. video consultations). Furthermore, the wider context of the coronavirus pandemic and timing of interviews (*n* = 13 conducted during/after first UK lockdown) could have influenced as some women were/had been shielding. We did not use a formal implementation framework, but the results fit the Consolidated Framework for Implementation Research [35], and future work may benefit from utilising a structured implementation approach.

## Conclusion

Limited previous research has explored ePRO follow-up instead of attending face-to-face follow-up. The findings of the complementary interviews presented here provide crucial information about women’s acceptability and experiences of this type of follow-up after OC treatment. This data illustrates that it is possible to build ePRO services that are acceptable and valued. This work should inform future development of ePRO follow-up pathways, highlights who may be suitable and emphasises the importance of building and resourcing ePRO services to meet the needs of patients to enable them to gain reassurance, confidence and live well post-treatment.

## Supplementary Information

Below is the link to the electronic supplementary material.

Supplementary file1 (DOCX 39 KB)

## Data Availability

The data that support the findings of this study are not publicly available due to privacy/ethical restrictions, but the corresponding author may consider reasonable requests.

## Abbreviations

ePRIME: (electronic Patient self-Reported outcomes to Improve cancer Management and patient Experiences)
ePRO: Electronic patient-reported outcome
OC: Ovarian cancer
PRO: Patient-reported outcomes
