# Supplementary material for: ‘It made me feel part of the team, having my homework to do’ — women and specialist nurse experiences of remote follow-up after ovarian cancer treatment: a qualitative interview study
Source: Support Care Cancer. 2022 Dec 13;31(1):2. doi: 10.1007/s00520-022-07470-z (PMC9745768; doi:10.1007/s00520-022-07470-z)
Supplement: Supplementary file 1 — Supplementary file1 (DOCX 39 KB) [file 520_2022_7470_MOESM1_ESM.docx]

*“It made me feel part of the team, having my homework to do”* – Women and specialist nurse experiences of remote follow up after ovarian cancer treatment: a qualitative interview study. Supportive Care in Cancer.

**Supplementary files**

**S1. Interview schedules**

**Women – general views**

The aim of the ePRIME study was to see whether asking patients to complete an online questionnaire every 3 months, have their bloods done locally and have a telephone review with a clinical nurse specialist is a suitable for monitoring patients.

- How have you found being on the ePRIME study?
- Were there any aspects of ePRIME that were helpful/unhelpful?

So thinking about the online questionnaire…

- How did you find using the ePRIME system? Was it easy to use?
- Did you have any difficulties finding the website, logging in, at any time on the system etc? If so, how did you resolve them?
- Did you use the user manual we gave you? If so, do you have any suggestions for how we might improve that? If not, why not?
- How did you feel about the questions that were asked in the online questionnaire? Did you find them relevant?
- Was there anything missing from the ePRIME online questionnaire?
- Were the reminders to complete the online questionnaire useful/not useful/right time?
- Did you ever request nurse contact? What happened?
- Did you have any problems with symptoms/side effects during ePRIME?
- Did you report them on the online system? Did you directly contact anyone about these? Who / How did you contact them? What happened following this contact/reporting your symptom on the system?
- Do you think you would’ve reported this to your clinical team if the ePRIME system hadn’t prompted you to?
- If not, why not? Did you take any other action, i.e. self-medicate/wait for routine appointment to discuss
- How long did it take you to complete the online questionnaire? Acceptable?

And thinking about having your blood test done locally…

- Where did you have your blood tests done?
- Were the blood test reminder text/emails useful/not useful/right time?
- Did you have any problems with obtaining blood tests (at the hospital/GP) or receiving the results?
- How did you feel about having your blood tests done (at the hospital/GP)?

And moving on to your telephone review appointments with the nurse…

- How did you feel about receiving follow up appointments over the telephone rather than face to face at a hospital? ((Note: some turned up instead – try to unpick why if happened – confusion /more convenient ?…))
- Do you think it had any effect on you/your follow-up care?
- How did you feel about telephone appointments being conducted by a nurse rather than a doctor?
- Did you encounter any problems with receiving telephone follow up?
- If you had a choice, would you want to continue with telephone follow up or return to hospital face-to-face follow up?
- What are the advantages of telephone follow up?
- What are the disadvantages of telephone follow up?

So thinking about the ePRIME follow up as a whole…

- Joined at X (CT scan results apt or later) – was that timescale OK? Earlier?
- What were your expectations of being on ePRIME follow up? Were your expectations met? Did ePRIME follow up meet your needs? Tell me more? Please elaborate?
- Do you think the CNS found the ePRIME information you provided online useful? Did you find completing the questionnaires online useful?
- You were reviewed 3 monthly – how was this frequency for you? (e.g. OK, too frequent, not frequent enough)
- Did you ever use the system more than 3 monthly? What prompted you to do this?
- Did you feel that ePRIME follow up had an impact on you? On your role in how you managed your symptoms? Did you find the advice provided at the end of the online system about your symptoms helpful? If so / if not why?
- Did ePRIME follow up change the nature of contact you had with clinical team?
- Thinking about how we can shape follow up services for women after ovarian cancer treatment in the future, what kind of follow up would you like to see?
- Would you be happy to use ePRIME follow-up again, now or in future?
- Would you recommend to other patients?
- What did your family/relatives think about you using the ePRIME system?
- Anything we could have done differently in ePRIME to make it work better for you?
- Do you have any other comments or questions about your involvement with ePRIME?

**Nursing staff questions - General experiences on study**

- Confirm involvement with ePRIME – recruitment of patients (?), responding to alerts, ePRIME reviews over the phone? Anything else?
- How did you find being involved with the ePRIME study?
- What were the advantages of ePRIME for you?
- What were the disadvantages of ePRIME for you?
- What were the advantages of ePRIME for your patients?
- What were the disadvantages of ePRIME for your patients?

So thinking first about the online symptom questionnaire that patients complete at home…

- How did you look at the Q results? On PROMPT or another way?
- How did you find viewing the results on PROMPT? How easy was it to access and find information you needed to review? How useful did you find the prompt guide (A4 sheet on navigating PROMPT)?
- Did you ever respond to an email alert from the system? If so can you talk me through any particular examples, what happened, what did you do when this happened (ring straightaway or wait for booked telephone appointment)? How could this alert system be improved? Were the alert levels about right, too sensitive?
- Do you think the online symptom Q captured everything it needed to? Anything missing?
- Were there any unexpected benefits or burdens of the online Q?
- Do you have any suggestions for how the system / questionnaire could be improved?
- Do you have any suggestions how we may improve communication with staff who are using the system?
- Do you think the Q encouraged any self-management of symptoms in patients?

## During ePRIME patients got a blood test done at a location at their preference (GP, hospital) …

- Were you aware of any issues with this process? Blood form/GP willingness/receiving results?

## Moving onto the telephone review appointments you conducted with ePRIME patients:

- How did you feel about providing follow up appointments over the telephone rather than face to face at a hospital? How did telephone FUP compare to hospital-based FUP?
- Did you encounter any difficulties with providing the ePRIME telephone FUP?
- Timing of ePRIME telephone FUP (being in line with questionnaire reminders)?
- Could you foresee any issues with moving over to telephone FUP in the future?
- Would you want to continue telephone FUP? (with or without the online Q component?)
- Did ePRIME follow up have any impact on the length or nature of your consultations?
- How useful was the information received via the online Qs?
- Were there any times when a patient reported symptoms in the consultation did not match the online Q symptoms? Could you give an example? What happened?
- Do you think ePRIME has any impact on the doctor/nurse-patient relationship?

Thinking about ovarian cancer follow up as a whole...

- Did you feel that ePRIME follow up had an impact on you? On your role as a CNS?
- Were there any aspects of ePRIME that were particularly helpful/unhelpful?
- Thinking about how we can shape follow up services for women after ovarian cancer treatment in the future, what kind of follow up would you like to see?

**S2. Table illustrating excerpt quotes for each theme and subtheme**

| **Seeking reassurance and confidence in remote follow-up** | |
| --- | --- |
| ***Theme 1: Readiness and motivators for remote follow-up*** | |
| **1.1. Motivators for joining** | 1. “I wanted to do it because for like, me and my parents talked before that the appointment system just not really working for us, like the amount of time we’d spend compared to like time within the room, so when we got suggested to this we thought it looks a really good idea for that, to stop having to drive all the way to [city] and sit in a waiting room and do all like over the phone.” (PT9)  2. “…they [consultant and CNS] were saying, “It’s a great system, we think it’s going to work”. I’ve got a lot of trust in them, and they were saying, and I think it’s true, compared with coming into the hospital, it’s less anxiety provoking, I noticed the difference.” (PT1)  3. “I kind of knew that I was in a fairly good place, sort of physically and emotionally with it all, and I was quite happy to just get on knowing that I’d got that sort of touch point. Like I said, I keep getting to a point so that it’s like, I’ve got nothing to report, so you just feel like you’re wasting a lot of people’s time when there’s other people who could probably do with a bit more time.” (PT11)  4. “I just wanted to help research and I thought if it can be a better system it would free up all the waiting time at the hospital as well, so it would be for a good thing really” (PT2)  5. “So that the resources were used in more, you know, critical and for people that weren’t well, you know, I’d rather give the time to them. I mean I am a retired nurse anyway so, you know, I did feel the need to use the resources where, you know, they’re sitting down with me, they could be doing something better.” (PT3) |
| **1.2. Patient readiness** | 6. “people like me, who are fairly symptom-free and sort of ready to move on, that it is a really good way of doing it.” (PT11)  7. “while I was well it worked perfectly for me. Because I’m actually a bit more remote, I’m based in [another city]…it meant that I didn’t have to travel for blood tests, I didn’t have to travel to actually see the oncologist.” (PT13 )  8. “Maybe within, not within the first year I don’t think, because I was obviously still having…scans done then, and I wanted to see the doctors to be able to, face-to-face, for them to reassure me, and reassure like my family that everything was normal and fine. But then the next like 2 years…the appointments became very routine, and it was like I’d be in the room for 5 minutes, everything’s fine, and then I’d leave, so it seemed like a lot of like waiting around for something that could be done a lot faster.” (PT9)  9. “I think that earlier would have been too soon, I wouldn’t have felt safe.” (PT4)  10. “personally I wouldn’t have minded doing it earlier.” (PT7) |
| ***Theme 2: Practicalities and logistics of remote follow-up*** | |
| - 1. **Individual patient factors** | 11. “I am used to doing, like using computers and things so it wasn’t a problem.” (PT5)  12. “Well, he [husband] got it, downloaded it and I just, well, he did say, “Answer this and I’ll press the button.” He pressed the button, whatever he did, and I just answered them…I’m not interested in computers or things like that.” (PT10)  13. “I think the only thing I think that it’s, for people like me that don’t show it through blood, you don’t have any back fall, you don’t have a safety net, at all, so I find giving blood completely useless, for me… so I think there should be something different for people like me that don’t show cancer through blood… For the people it doesn’t show you actually have no confidence in that, because you can’t trust your own body.” (PT4)  14. “I’m going to hate myself for saying this, but I think that’s probably about the stage that I was at originally and going onto ePRIME, or the stage what I was originally and going onto six month reviews at any point, because I was advanced, I was stage 3C. And I know there is some debates about this kind of review and women with advanced ovarian cancer, I know that there’s some resistance to advanced going on it. And I’ve always said no, because you wouldn’t let me on it if, and, actually, I’m really enjoying it, but I think, I’ve a feeling now that they might be right.” (PT13) |
| - 1. **The technical aspects** | 15. “It was very easy, it was really, really easy, and they also gave sections where you could actually put in what you wanted, which was really useful because obviously sometimes ticking a box doesn’t quite say everything, but if you’ve got a place to put, add to that, then that was perfect, I thought it was a very easy system to use.” (PT4)  16. “I found it very convenient and very easy to log on and complete the questionnaire.” (PT7)  17. “There was the unfortunate first time, when I had some symptoms, it worried me when the system had crashed. It was just, you know, one of those things really, and I felt quite disconcerted about that…That was scary, and I remember speaking to one of the nurse specialist teams, and they just said, “It’s okay, we’ll speak to you” (PT1)  18. “[reminders] seemed to be out of sync, you know receiving the reminders when it wasn’t coming up to an appointment.” (PT5)  19. “No I don’t think, no I don’t think I have had any reminders” (PT14)  20. “I think because it was so ad-hoc, you forgot how to do it. So every time you went into it, it did feel as if ‘oh how did I do it?’ but it’s, you soon remembered how to do it and yeah, that were quite, that were pretty, I liked that because like you say you could actually input your outcome on it…if that then could be linked into our EPR system, we don’t really then have to do separate documentations” (Nurse 2)  21. “I think being in [name of EPR] made it a huge amount easier, much easier…But once you got to where you were going it was fine. And I found the graphs and everything really helpful.” (Nurse 4) |
| - 1. **The value of the core features** | *ePRO questionnaire*  22. “I actually prefer it. I do on the, they seem to be a bit more relevant because of the questions on the questionnaire” (PT5)  23. “It was my go-to thing, you know, “Right, I’m going to fill in my questionnaire and I’m going to phone”, that’s what ended up doing.” (PT1)  24. “I did have, or still do have, just a couple of little niggles…If I’d been face-to-face with either the consultant or the keyworker I would have probably have mentioned it, but there was nowhere on the questionnaire that I could express it.” (PT3)  25. “Yeah and the added benefit of that is when I’m looking at that tool that they’ve just filled in, I’m thinking, I’m thinking before you ring that patient you’ve got a plan in your head, you’ve got some suggestions you can make, you’re not on the hoof. I mean you know, we should be skilled to do it on the hoof and we are, but it’s just more smoother, more concise” (Nurse 4)  26. “I think we probably don’t need the questionnaire for things like that because if you’re going to ring them, as long as they have their bloods done, if they’re going to ring them anyway, we probably don’t need that questionnaire” (Nurse 2)  *Alerts and advice*  27. “I think the only thing was obviously one of the questions it kept asking me and telling me that I needed to contact the hospital because of…but I did write at the bottom of every time it asked that question that it’s normal for me, I know it might look like a problem but it’s normal for me so I didn’t need to follow it up like it kept alerting me that I needed to.” (PT5)  28. “So I’m putting in, under the question about abdomen being swollen, I’ve got a swollen abdomen, and then that was telling me to ring the nurses, you know, and so I was doing it, and then they were saying, “No, but we know that”, there was a bit of confusion. So I stopped saying I had one even though I had.” (PT1)  29. “Oh yes, it did, it came up with…like a remedy thing, and of you know, maybe yeah, oh have you, I mean that could be just a lack of iron, or it said to rest and things like that, it did give you, it did give you information of what it could be, which was helpful, yes.” (PT12)  30. “I could see how it would be useful for anybody that were perhaps not as knowledgeable as me.” (PT13)  31. “I think that’s another thing we felt, it felt that we had to [respond to alerts], but that’s us, that’s very much us. I would say well we don’t have to, they know how to contact us, they’re flashed up to contact us, don’t worry about it.” (Nurse 4)  32. “So yes ideally, that was the main thing wasn’t it, wait until the clinic appointment, because we’ve made that clinic appointment. But say if the timescale was different and there was a bit of a longer period between, and they’d generated a concern, then they would get a phone call and then it might be that we didn’t do the appointment the week after.” (Nurse 2)  *Access to blood tests*  33. “I had to say, was, “I’m being monitored by [hospital], they want me to have my cancer markers done. Can I have an appointment, I’ll bring my own form”, and they said yes. So it all just worked very well.” (PT1)  34. “I came to the hospital er…probably once, and then I started to, unfortunately, for some very odd reason, my GP practice doesn’t do blood tests there, so I went to the local hospital, which is [name of local hospital].” (PT7)  35. “getting the bloods out was tricky, but of course now we’ve got electronic bloods for most of [Hospital], so we used to send out an envelope in the post to them, so they’d toddle along with, now we just put them on the system live… before the secretary used to ring down and say, I’m doing these letters, can I have some blood forms, she’d ring the next day and say you’ve not done them yet, come on, I want my blood forms, I think all those little things, like purely coincidences have been smoothed out.” (Nurse 4)  *Telephone review appointments*  36. “I do get anxious as soon as, I know the number because it ends in treble 9, so I know the hospital number so as soon as that comes up I think, you know, gosh, this is the hospital and I’m so anxious to know what my blood results are. But they are reassuring, they are reassuring the nurses, they’re all good really that’s phoned me, and you know, and kind of calm me down and say, look, you know, don’t panic, it’s fine…And they ask how you are and everything. So I prefer that than as I say going, anything to keep me out of hospital really *[laughs]*, just not wanting to go to the hospital.” (PT12)  37. “I think the online survey could be used to decide whether people need telephone appointments, like that for example for me if I’ve not reported any symptoms I suppose I don’t really need a telephone appointment, because I was just saying, yeah, I’m fine. **Researcher: Yeah…I’m just thinking about whether you got anything more from that telephone appointment that you wouldn’t have if you didn’t speak to the nurse, in terms of the result of the blood test?** Oh yeah, I would have got that at the telephone appointment…and the reassurance that the team is still in contact with you I suppose, not forgotten about…” (PT9) |
| - 1. **Engagement and integration with the clinical service** | 38. “…there always seemed to be somebody were booked on wrong, or they weren’t booked in where they should be, they weren’t on the schedule, because they set up a certain schedule, didn’t they, erm…, you know, so their appointments had to fit a certain timeframe, didn’t they?” (Nurse 1)  39. “very little negatives in it, more admin, remembering to put them on the right code, things like that, so sometimes a patient would turn up, they’ve got the wrong letter, and getting the bloods out was tricky” (Nurse 4)  40. “And the fact that there was always somebody on-hand, particularly in the beginning…“can someone tell me how to find this report again?”, [laughs], so I think the fact that we had good working relationships was a real key point to that study working well.” (Nurse 4)  41. “if we said right, this type of follow-up is going to be for all our patients that can be on remote follow-up, I actually think it would work better. And you’d make it work and you’d get it used to it, instead of just having an odd one here and an odd one there”. (Nurse 2) |
| ***Theme 3: Personal impact*** | |
| **3.1 Anxiety levels** | 42. “It’s always quite daunting to go to the hospital because it holds, you know, lots of quite scary memories.” (PT7)  43. “one lady said to me, you know, I used to get up on a morning and think, oh I’m going to that hospital for this, used to get my best clothes out of the wardrobe because I’m coming to see the doctor or the nurse, and then I panic about parking, and then I get into the hospital, I sit two hours, and I’m worried that you’re going to tell me something. She said this way, she said I’m in the middle of doing my tapestry, I’m at a tricky point, I don’t really want to talk to you right now and I’m fine. So for them I think there’s, it lessens anxiety, and I think they get a better quality consultation” (Nurse 4)  44. “I like the idea of filling out the questionnaire first because I think things then aren’t missed, because I don’t know about most people but when I go to a hospital for an appointment, you go in there and you come out and you think, ‘oh I forgot to tell them this and I forgot…’ but with having it, a questionnaire in your own home when you’re relaxed and you, you know, you’re not going to miss things, well you might but it’s less likely, it’s less likely.” (PT5) |
| **3.2 Connection/safety** | 45. “yeah, it didn’t make me feel I was being put at a distance by it, it didn’t feel like any kind of, “Oh, because of this I haven’t got the same connection with the doctor”, etc.” (PT1)  46. “I know I’ve got to keep my eyes open and there is a good chance that it will come back, but it just doesn’t feel like the dominating thing in your life any more, it’s just something, you know. I don’t want to trivialise it, but it just feels like, you know, I just…go into my study, I’m doing my emails, “Oh, there’s the one from ePRIME”, and I fill it in and, yeah, onto the next thing and it’s not, but I do feel that somebody’s sort of looking after me.” (PT11)  47. “I think, I don’t know, there is a lack of something when you’re not face-to-face with somebody, like speaking to you really. I don’t know if we could have FaceTimed or Zoomed or whatever but it’s just nice to talk to somebody face-to-face really.” (PT3) |
| **3.3 Patient empowerment/self-efficacy** | 48. “I think it has shown that patients are more than responsible, are more than capable…I think it has made the health professional give the patient control back, because we do, we struggle to give them control back, and it’s something that I’m always saying to my team, you know, oh Mrs So and So wasn’t very well last weekend and we didn’t know, and oh, and yes but she could have rung us, where we always feel the guilt that it’s us that should have been checking up on them or whatever. So I think it has really empowered the patient, because it’s made the health professional take a step back and we’re not good at that.” (Nurse 4) |
| **3.4 Time and resources** | 49. “I could see a stretch of symptoms and the variation of those symptoms over time. And that’s really helpful…having that availability of that snapshot was quick, was concise, it was a really good overview, and also helped shorten the consultations.” (Nurse 4)  50. “with this system I find there’s plenty of time in the slot to have a read, get everybody up to speed, open your graphs, have a look at everything and ring the patient. And I just think they get a better quality consultation in that way.” (Nurse 4)  51. I think it ended up being more work…Not for me as such, but, yeah, you know, obviously when stuff was coming back and then they were getting problems and then it was like, some of them weren’t on the right clinic and then they weren’t always straightforward.” (Nurse 1) |
| ***Theme 4: Future role of remote follow-up*** | |
| **4.1. Future use** | 52. “Strangely, I’d like, if I had the choice, I would continue on ePRIME…Strangely, because, you know, I’ve now got my three monthly appointment system back and that ought to make me feel, that’s what I’d prefer that, you know, more contact with the hospital. But maybe it’s not strangely, as I say, no, it’s quite anxiety provoking, coming in.” (PT1)  53. “I’ve been really pleased with it. I’d be quite sad if it went away.” (PT11)  54. “I’d be very supportive of rolling it out. Definitely, and if you could get that little prompt for me in my nurse-led clinic I’d be very pleased [laughs].” (Nurse 4)  55. “I think with some tweaking, it could work to [small pause] help with like the clinic, the discussion in clinic and that document is generated as a GP letter… if we’re now widening our remote follow-up of patients, weren’t, actually this now potentially could be rolled out, in my eyes, to everybody who’s able to have a telephone consultation. Whereas that weren’t happening before. So there’s probably more patients having telephone consultations, so actually this could be rolled out as a, like you just said, fill this in two days before you come into clinic and it will guide, you know.” (Nurse 2) |
| **4.2. Suggestions for improvements** | 56. “The video chat was the only thing that I thought would actually make a massive improvement to a lot of people” (PT4)  57. “And, as I say, the only thing that I have slight problems with were just a little bit of breakdown with the blood test results, getting them across to [main hospital], but that’s just, unfortunately, because you have to go through so many different channels.” (PT7)  58. “I do think if it’s, if this is the way that, if this programme gets rolled out further, it probably does need a bit of a prod at some GPs” (PT11)  59. “Well I think the opportunity as I said before, you know, to make little comments because I think to a certain extent it is guidance, you know. I mean you see my weight does fluctuate but that’s nothing to do with the cancer, absolutely nothing to do with the cancer, you know. I just hate cooking, you know, absolutely hate it.” (PT6)  60. “I think perhaps if there is a very short cover note with the envelope, so that when I go in to see the practice nurse that explains why I’m having it done there.” (PT13) |
